# The role of inflammation in the prospective associations between early childhood sleep problems and ADHD at 10 years: findings from a UK birth cohort study

**DOI:** 10.1111/jcpp.13755

**Published:** 2023-01-03

**Authors:** Isabel Morales‐Muñoz, Rachel Upthegrove, Kate Lawrence, Rasiah Thayakaran, Sandra Kooij, Alice M Gregory, Steven Marwaha

**Affiliations:** ^1^ Institute for Mental Health University of Birmingham Birmingham UK; ^2^ Early Intervention Service, Birmingham Women's and Children's NHS Trust Birmingham UK; ^3^ Department of Psychology St Mary's University Twickenham London London UK; ^4^ Institute of Applied Health Research University of Birmingham Birmingham UK; ^5^ Department of Psychiatry, Amsterdam Public Health Research Institute VU University Medical Center Amsterdam The Netherlands; ^6^ PsyQ, Expertise Center Adult ADHD The Hague The Netherlands; ^7^ Department of Psychology, Goldsmiths University of London London UK; ^8^ Specialist Mood Disorders Clinic Zinnia Centre Birmingham UK; ^9^ The Barberry National Centre for Mental Health Birmingham UK

**Keywords:** Sleep, ADHD, inflammatory markers, ALSPAC, longitudinal

## Abstract

**Background:**

Several underlying mechanisms potentially account for the link between sleep and attention deficit and hyperactivity disorder (ADHD), including inflammation. However, studies so far have been cross sectional. We investigate (a) the association between early childhood sleep and probable ADHD diagnosis in childhood and (b) whether childhood circulating inflammatory markers mediate these prospective associations.

**Methods:**

Data from the Avon Longitudinal Study of Parents and Children were available for 7,658 10‐year‐old children. Parent‐reported sleep duration, night awakening frequency and regular sleep routines were collected at 3.5 years. The Development and Wellbeing Assessment was administered to capture children with clinically relevant ADHD symptoms, or probable ADHD diagnosis. Blood samples were collected at 9 years, from which two inflammatory markers were obtained [interleukin‐6 (IL‐6) and C‐reactive protein (CRP)]. Logistic regression analyses were applied to investigate the associations between sleep variables at 3.5 years and probable ADHD diagnosis at 10 years. Further, path analysis was applied to examine the potential mediating role of inflammation at 9 years (as measured by CRP and IL‐6) in the associations between early sleep and ADHD at 10 years.

**Results:**

Less regular sleep routines (OR = 0.51, 95% CI = 0.28–0.93, *p* = .029), shorter nighttime sleep (OR = 0.70, 95% CI = 0.56–0.89, *p* = .004) and higher night awakening frequency (OR = 1.27, 95% CI = 1.06–1.52, *p* = .009) at 3.5 years were associated with higher odds of ADHD at 10 years. Further, IL‐6 at 9 years, but not CRP, mediated the association between irregular sleep routines and ADHD (bias‐corrected estimate, −0.002; *p* = .005) and between night awakening and ADHD (bias‐corrected estimate, 0.002; *p* = .003).

**Conclusions:**

Several sleep problems in early childhood constitute a risk factor for probable ADHD diagnosis at 10 years. Further, these associations are partially mediated by IL‐6 at 9 years. These results open a new research vista to the pathophysiology of ADHD and highlight sleep and inflammation as potential preventative targets for ADHD.

## Introduction

A good night of sleep is essential for every aspect of a child's functioning (Mindell et al., 2011). Further, identification of sleep problems in children is important because they are associated with physical, cognitive and socio‐emotional problems (Bayes & Bullock, 2020; Virring, Lambek, Thomsen, Moller, & Jennum, 2016). For instance, sleep problems are commonly comorbid with neurodevelopmental conditions, including attention deficit and hyperactivity disorder (ADHD) (Bondopadhyay, Diaz‐Orueta, & Coogan, 2022). Systematic reviews indicate that the prevalence of ADHD in children is around 5% (Sayal, Prasad, Daley, Ford, & Coghill, 2018) and is characterized by attention difficulties, impulsivity and/or hyperactivity (American Psychiatric Association, 2013). The aetiology of ADHD is multifactorial and includes both genetic and environmental factors (Banerjee, Middleton, & Faraone, 2007; Faraone et al., 2005; Lewis et al., 2022). One manifestation of this aetiology is disturbed sleep, and there has been a dramatic increase in new research to better understand the association between sleep and ADHD (Weiss, Craig, Davies, Schibuk, & Stein, 2015).

Sleep disturbances are commonly comorbid with ADHD, and between 70% and 85% of children with ADHD experience sleep problems (Yürümez & Kılıç, 2013). Sleep problems, such as fragmented sleep, bedtime resistance and/or sleep‐onset insomnia in early childhood, precede ADHD symptoms. For instance, sleep disturbances at age 4 associate with attention problems at age 15 (Gregory & O'Connor, 2002). Other studies have revealed similar associations at earlier stages, with sleep problems between 2 and 4 years old being associated with attention deficits at age 5 (O'Callaghan et al., 2010), or short sleep duration from 3 to 24 months associating with inattention at 5 years (Huhdanpää et al., 2019). These studies suggest that sleep problems in early childhood may be an initial symptom, risk indicator of later ADHD. However, most of these longitudinal studies have focused on ADHD symptomatology, rather than ADHD diagnosis. Further, it is still unclear whether all or only some specific sleep problems in early childhood precede ADHD, although previous reviews and meta‐analyses suggest that different sleep variables are associated with ADHD (Cortese, Faraone, Konofal, & Lecendreux, 2009; Gregory & Sadeh, 2012). Further, sleep problems in ADHD can result in significant functional impairments affecting mood, behaviour and quality of life (Wajszilber, Santiseban, & Gruber, 2018), which supports the necessity of further investigating sleep problems in ADHD.

If sleep and ADHD are robustly and prospectively associated, the next step would be to understand the potential mechanisms by which sleep difficulties may increase the risk for ADHD. Among these potential candidates, inflammation has recently received some attention, and preliminary evidence suggests that inflammatory processes could contribute to the association between sleep and ADHD (Wynchank, Bijlenga, & Kooij, 2018), although this requires further research consideration.

Sleep disturbances have been associated with altered levels of inflammatory cytokines in adults (Irwin, Olmstead, & Carroll, 2016), but data in youth are still lacking. Two of the inflammatory markers that have received greatest attention in relation to sleep are interleukin‐6 (IL‐6) and C‐reactive protein (CRP) (Irwin et al., 2016). IL‐6 is a proinflammatory cytokine produced in response to environmental stressors, infections and injuries. Dysregulated and persistent IL‐6 production leads to the development of various diseases (Tanaka, Narazaki, & Kishimoto, 2014). CRP is a pentameric protein synthesized by the liver, and its level rises in response to inflammation (Nehring, Goyal, & Patel, 2022). A recent study reported that morning serum IL‐6 associates with actigraphy‐based sleep efficiency and time awake in young people (LaVoy, Palmer, So, & Alfano, 2020). Further, another study reported higher CRP and IL‐6 levels in children with ADHD, compared to healthy children (Chang et al., 2020; Darwish, Elgohary, & Nosair, 2019). Currently, as far as we are aware there is no research which has specifically investigated the prospective association between inflammation, sleep and ADHD. Increased understanding here would allow us to further interrogate potential mechanisms underlying the links between disturbed sleep and ADHD and potentially design better targeted early interventions in ADHD.

Therefore, the aims of this study were to investigate (a) the association between sleep variables in early childhood and probable ADHD diagnosis in childhood and (b) whether circulating markers of inflammation (i.e., CRP and IL‐6) in childhood mediate the associations between sleep variables and later probable ADHD diagnosis. We hypothesized that shorter nighttime sleep duration would be the key sleep variable associated with later ADHD (Huhdanpää et al., 2019), and that CRP and IL‐6 would mediate the prospective association between sleep and ADHD.

## Methods and materials

### Participants

The Avon Longitudinal Study of Parents and Children (ALSPAC) is a UK birth cohort study examining the determinants of development, health and disease during childhood and beyond (Boyd et al., 2013; Fraser et al., 2013). 14,541 pregnant women resident in Avon, UK, with expected dates of delivery 1 April 1991 to 31 December 1992 were invited to take part in the study. A flowchart describing the population selection for this study appears in the Supporting Information (Figure S1). The ALSPAC study website contains details of all the data available through a fully searchable data dictionary and variable search tool (http://www.bristol.ac.uk/alspac/researchers/our‐data/). Further details of the ALSPAC are provided in the Supporting Information (Appendix S1). Informed consent for the use of data collected via questionnaires and clinics was obtained from participants following the recommendations of the ALSPAC Ethics and Law Committee at the time. Ethical approval was obtained from the ALSPAC Law and Ethics committee and the local research ethics committees.

### Measures

#### Sleep assessment at 3.5 years old

Parent‐reported sleep routines regularity (i.e., ‘Does your child have regular sleep routines’), daytime sleep duration (i.e., ‘How long does your child sleep during the day?’), nighttime sleep duration and night awakenings frequency (i.e., ‘How often during the night does your child usually wake?’) were assessed when the child was 3.5 years old. Nighttime sleep duration was calculated from questions asking what time (to the nearest minute) the child ‘normally’ went to sleep in the evening (bedtime) and woke up in the morning (waking time). Parent‐reported sleep routines regularity was a dichotomous variable (i.e., Yes/No), while the other three parent‐reported sleep variables were continuous. This time point was focused upon based on previous findings that it showed the strongest prospective associations with mental health problems in adolescence (Morales‐Muñoz, Broome, & Marwaha, 2020). Further, by the age of 3, children's sleep–wake patterns are consolidated (Paavonen et al., 2020).

#### Probable ADHD diagnosis at 10 years old

Probable ADHD diagnosis at 10 years old was assessed via parental ratings, using the Development and Well‐Being Assessment interview (DAWBA) (Goodman, Ford, Richards, Gatward, & Meltzer, 2000). Further details of the DAWBA are provided in the Supporting Information (Appendix S2). Considering that most cases with ADHD are diagnosed when children are 6–11 years old (Wilens & Spencer, 2010), we focused on ADHD at 10 years to make sure that the majority of the potential cases with ADHD would be characterized. DAWBA was administered via computer, generating the following “probability bands”: 0: <0.1% probability of children in this band having the disorder; 1: ~0.5%; 2: ~3%; 3: ~15%; 4: ~50%; 5: >70%, respectively. The top two levels of the DAWBA bands were used as computer‐generated DAWBA diagnoses, which was the measure selected for this study. This follows previous studies using the DAWBA bands, which were validated in 7,912 British children and 1,364 Norwegian children, using clinician‐rated DAWBA diagnoses as a gold standard (Goodman, Heiervang, Collishaw, & Goodman, 2011). Briefly, the prevalence estimates of the computer‐generated DAWBA diagnoses were of roughly comparable magnitude to the prevalence estimates from the clinician‐generated diagnoses, but the estimates were not always very close. In contrast, the estimated effect sizes, significance levels and substantive conclusions regarding risk factor associations were very similar or identical. Therefore, the computer‐generated DAWBA diagnoses may provide a useful alternative to clinician‐rated diagnoses, when studying associations with risk factors, generating rough prevalence estimates or implementing routine mental health screening. Therefore, here we use the term ‘probable ADHD diagnosis’, instead of ADHD diagnosis.

#### Inflammatory markers at 9 years old

Blood samples were collected from non‐fasting participants at 9 years. Samples were immediately spun, frozen and stored at −80°C. High‐sensitivity CRP (hsCRP) was measured at one time point at the same laboratory by automated particle‐enhanced immunoturbidimetric assay (Roche UK, Welwyn Garden City, UK). Additionally, IL‐6 was measured by single enzyme‐linked immunosorbent assay (R&D Systems). All assay coefficients of variation were <5%. Data on IL‐6 and CRP were available for 4,223 and 4,232 participants, respectively. In the total sample, CRP values ranged from 0.01 to 67.74 mg/L. Fifty‐nine subjects had CRP levels >10 mg/L and were excluded due to the risk of confounding by acute inflammatory state. IL‐6 values ranged from 0.01 to 20.05 pg/ml and all values were kept for the analyses. Higher levels of IL‐6 (Tanaka et al., 2014) and CRP (Landry, Docherty, Ouellette, & Cartier, 2017) are both associated with higher probability of infection. IL‐6 and CRP levels were log‐transformed and standardized (Z‐transformed), following previous research (Perry, Zammit, Jones, & Khandaker, 2021).

#### Confounders

Multiple family risk factors were assessed using the Family Adversity Index (FAI) during pregnancy, and at 2 and 4 years. The FAI has been developed by the ALSPAC study team (Steer & Wolke, 2004) and comprises 18 items on early parenthood, housing conditions, maternal education, financial difficulties, parents' relationship, family size, family major problems, maternal psychopathology, parents' substance abuse, crime records, partner support and social network. More specifically, the items used in the FAI were (the number of items per block are presented in brackets): *Age of mother younger than 20 years at first pregnancy*/*child birth* (1 items); *Housing* (3 items): (a) inadequacy: crowding index/periods of homelessness; (b) Basic living: no availability of hot water or no indoor toilet, bath or shower or no kitchen (c) major defects/infestation; *No educational qualifications* (*mother or father*) (1 item); *Financial difficulties* (1 item); *Partner relationship* (4 items) (a) status, (b) affection and aggression, (c) physical /emotional cruelty, (d) no social support; *Family* (2 items): (a) family size (>3 children), (b) Major care giving problems (child in care /not with natural mother, or on social services at risk register); *Social network*: (a) no emotional support, (b) no practical /financial support (2 items); *Maternal affective disorder* (*Depression, anxiety, suicidality*) (1 item); *Substance abuse* (1 item): (a) drugs or alcohol (use of hard drugs, alcoholism, high alcohol consumption); *Crime* (2 items): (a) in trouble with police or (b) convictions. Each individual item is assigned a value of 1 if an adversity is present and 0 if it is not present; hence, the FAI has a range of 0–18. FAI scores were calculated where more than half of the items were valid, and non‐adversity was assumed for any missing data, following previous research (Collin et al., 2015; Crawley et al., 2012). Points were summed at each time point, and finally the three time points were summed (i.e., pregnancy +2 years +4 years), to create a total FAI score, which was the variable used for this study. A total FAI score was not calculated for those individuals with no assessment available at one or more of the three time points. Higher scores represent higher number of early life family adversities. We included this variable as confounder, as adverse family environment is associated with ADHD (Thapar, Cooper, Jefferies, & Stergiakouli, 2012).

Other socio‐economic factors selected as covariates were child's sex, preterm delivery, ethnicity (white vs non‐white), maternal age when baby was born and maternal socio‐economic status, which was measured using the Cambridge Social Interaction and Stratification Scale‐CAMSIS (Prandy & Jones, 2001). The CAMSIS measures occupational structure based upon social interactions, with possible scores ranging between 1 (least advantaged) and 99 (most advantaged); thus, higher scores indicate more advantaged socio‐economic status. All these confounders were selected as there is a higher prevalence of ADHD in males (Rucklidge, 2010), preterm children (Montagna et al., 2020), white children (Shi et al., 2021), children with young mothers (Chang et al., 2014) and low socio‐economic status (Russell, Ford, Williams, & Russell, 2016).

### Statistical analysis

We first ran weighted logistic regression analyses in SPSS‐v27, to ascertain the unadjusted and adjusted associations between sleep variables at 3.5 years and computer‐generated DAWBA diagnoses of ADHD at 10 years. In Model A, we tested unadjusted associations. In Model B, we controlled for all confounders. Further, the four sleep variables were evaluated together in the same model, to account for the potential overlap between sleep patterns in childhood. As 50.3% of the original sample was lost to attrition by the 10‐year follow‐up, we conducted logistic regressions to identify significant predictors of attrition. Children lost due to attrition were more frequently born preterm and more often of non‐white ethnicity and had lower birth weight, shorter gestational age, younger mothers and higher FAI scores (see Table S1). Using the variables associated with selective dropout as the predictors, we fitted a logistic regression model (nonresponse vs response outcome) to determine weights for each individual using the inverse probability of response. The regression coefficients from this model were used to determine probability weights for the covariates in the main analyses. Therefore, the inverse probability weighting method was applied to the logistic regression analyses, to account for attrition.

To examine the mediating role of inflammation at 9 years, mediation models were tested using path analysis in SPSS‐Amos v27, with maximum likelihood estimation to test the effect of sleep variables at 3.5 years on computer‐generated DAWBA diagnoses of ADHD at 10 years, with inflammatory markers at 9 years as mediators. We also controlled for the potential associations between the explanatory variables (i.e., sleep variables). IL‐6 and CRP were tested separately in independent mediation models. We included as explanatory variables those sleep variables with significant associations in Model B, from the regression analyses. We also controlled for sex, FAI and preterm delivery, as these were the significant covariates from the regression analyses. We used bootstrapped bias‐corrected confidence intervals and *p* values for assessing the significance of the standardized direct, indirect and total effects. Missing data were imputed using the full information maximum likelihood method, and it represented 53.43% of the data (i.e., 7,769 available cases at 10 years, out of a total of 14,541 initial cases).

Finally, sensitivity analyses using propensity matching score (PMS) were applied, to ascertain whether the findings represented true effects or whether they were mainly due to residual confounding. PMS is a technique that simulates an experimental setting in an observational data set and creates a treatment group and a control group from the sample (Rosenbaum & Rubin, 1983). This way, PMS controls more effectively than regression approaches for the effects of observed confounders, such that while results remain observational, bias attributable to confounding reduces significantly. ‘Exposed’ (i.e., probable ADHD diagnosis at 10 years) and ‘unexposed’ (i.e., non‐probable ADHD diagnosis at 10 years) sets were matched on a set of variables that would otherwise confound comparisons between them. Therefore, propensity scores were developed accounting for all relevant available factors potentially accounting for the associations between sleep and probable ADHD diagnosis. Accordingly, individual propensity scores were calculated through logistic regression modelling based on the following 8 covariates: sex, FAI, preterm delivery, ethnicity, maternal age at child's birth, maternal socio‐economic status, maternal tobacco consumption during pregnancy and child's intelligence quotient. The ‘unexposed’ and ‘exposed’ cases were then paired 5: 1 (i.e., ratio of 5 unexposed to one exposed) nearest neighbour matching.

## Results

Data were available on 7,769 participants whose mothers reported on ADHD information at 10 years. Table 1 shows the frequencies and descriptive values of socio‐demographic, sleep and clinical variables. A description of these variables in children with probable ADHD diagnosis, compared to non‐ADHD are presented in Table 2. Further, correlations between the sleep variables are provided in Table S2. Briefly, all the significant correlations were weak (i.e., <0.2) or moderate (i.e., 0.2–0.4), which supports the inclusion of all the sleep variables within the same model, as these low values did not suggest any potential multicollinearity.

**Table 1:** Descriptive variables of the sample at 10 years old

	Frequencies		Descriptives	
	*N*	%	Mean	*SD*
Socio‐demographic factors				
Sex (boys/girls) (*N* = 7,769)	3,919/3,850	50.4/49.6	—	—
Ethnicity (white/non‐whites) (*N* = 7,198)	7,077/121	98.3/1.7	—	—
Birth weight, grammes (*N* = 7,333)	—	—	3,420.01	542.77
FAI, total score (*N* = 7,005)	—	—	3.93	4.01
Maternal age when birth (*N* = 7,429)	—	—	29.05	4.58
Gestational age, weeks (*N* = 7,429)	—	—	39.45	1.85
Preterm delivery (Y/N) (4516)	347/4,169	7.7/92.3	—	—
Maternal socio‐economic status (*N* = 6,466)	—	—	54.95	13.54
Sleep variables at 3.5 years				
Regular sleep routines (Y/N) (*N* = 6,860)	6,395/465	93.2/6.8	—	—
Daytime sleep duration, hr (*N* = 6,831)	—	—	0.35	0.70
Nighttime sleep duration, hr (*N* = 6,801)	—	—	10.73	0.84
Night awakenings per night, *n* (*N* = 6,824)	—	—	0.57	0.82
ADHD at 10 years				
DAWBA probable ADHD diagnosis (Y/N) (*N* = 7,769)	128/7,641	1.6/98.4	—	—
Inflamatory markers at 9 years				
CRP mg/L (*N* = 4,232)	—	—	0.55	0.21
IL‐6 pg/ml (*N* = 4,223)	—	—	1.28	1.57

CRP values ranged from 0.01 to 10 mg/L; and IL‐6 values ranged from 0.01 to 20.05 pg/ml. ADHD, Attention deficit and hyperactivity disorder; CRP, C‐reactive protein; DAWBA, Development and Well‐Being Assessment interview; FAI = Family adversity index; IL‐6, Interleukin 6.

**Table 2:** Differences in socio‐demographic and sleep variables between children with and without probable ADHD diagnosis at 10 years

Variables	Probable ADHD diagnosis at 10 years		
	Yes (*n* = 128)	No (*n* = 7,641)	*p*
	*N* (%)	*N* (%)	
Sex			
Male	104 (2.7)	3,815 (97.3)	<.001
Female	24 (0.6)	3,826 (99.4)	
Preterm delivery			
Yes	15 (4.3)	332 (95.7)	<.001
No	70 (1.7)	4,099 (98.3)	
Regular sleep routines 3.5 years			
Yes	96 (1.5)	6,299 (98.5)	.004
No	15 (3.2)	450 (96.8)	
Ethnicity			
White	112 (1.6)	6,965 (98.4)	.951
Other	2 (1.7)	119 (98.3)	
	**Mean (*SD*)**	**Mean (*SD*)**	***p***
Maternal age when born (years)	28.41 (5.41)	29.06 (4.57)	.117
Family Adversity score	7.19 (5.47)^a^	3.87 (3.96)	<.001
Gestational age (weeks)	39.02 (1.92)	39.45 (1.85)	.011
Maternal socio‐economic status	49.91 (13.27)	55.03 (13.53)	<.001
Birth weight, kg	3.31 (0.55)	3.42 (0.54)	.035
Daytime sleep duration, hr	0.52 (0.87)	0.35 (0.70)	.010
Nighttime sleep duration, hr	10.83 (1.18)	10.73 (0.83)	.187
Night awakenings per night, *n*	0.84 (1.22)	0.57 (0.81)	.001
CRP mg/L	0.52 (0.86)^a^	0.56 (1.02)	.786
IL‐6	1.36 (1.37)^a^	1.28 (1.57)	.707

CRP, C‐reactive protein; IL‐6, Interleukin 6.

^a^ Children with probable ADHD diagnosis at 10 years old reported significant higher score in Family Adversity Index (i.e. higher frequency of family adversities in early childhood) compared to children with no diagnosis of ADHD. There were not statistically significant differences between ADHD and non‐ADHD children for CRP and IL‐6 levels; however, children with probable ADHD diagnosis at 10 years presented slightly lower CRP levels at 9 years, while higher IL‐6 levels at 9 years.

The associations from the logistic regressions between sleep at 3.5 years and probable ADHD diagnosis at 10 years appear in Table 3. In Model A, the four sleep variables at 3.5 years old were significantly associated with probable ADHD diagnosis at 10 years. In Model B, children with less regular sleep routines (OR = 0.51, 95%CI = 0.28–0.93, *p* = .029), children with shorter nighttime sleep duration (OR = 0.70, 95%CI = 0.56–0.89, *p* = .004) and children with higher night awakening frequency (OR = 1.27, 95I%CI = 1.06–1.52, *p* = .009) had higher odds of probable ADHD diagnosis.

**Table 3:** Logistic regression analyses between sleep problems at 3.5 years and probable ADHD diagnosis at 10 years

Probable ADHD diagnosis at 10 years						
	Unadjusted model (Model A)			Adjusted model (Model B)		
	OR	CI 95%	*p*	OR	CI 95%	*p*
Regular sleep routines, 3.5 years	**.35**	**.20–.63**	**<.001**	**.51**	**.28–.93**	**.029**
Daytime sleep duration, hr, 3.5 years	**1.38**	**1.08–1.76**	**.009**	1.24	.97–1.58	.088
Nighttime sleep duration, hr, 3.5 years	**.73**	**.57–.92**	**.007**	**.70**	**.56–.89**	**.004**
Night awakenings per night, 3.5 years	**1.29**	**1.09–1.53**	**.003**	**1.27**	**1.06–1.52**	**.009**
Sex	—	—	—	**.29**	**.18–.47**	**<.001**
Maternal age birth	—	—	—	1.01	.97–1.05	.712
FAI	—	—	—	**1.12**	**1.08–1.16**	**<.001**
Ethnicity	—	—	—	1.32	.43–4.06	.630
Preterm	—	—	—	**.30**	**.18–.50**	**<.001**
Socio‐economic group	—	—	—	1.02	.94–1.12	.584

FAI, Family adversity index; it comprises 18 items (i.e., long index) on early parenthood, housing conditions, maternal education, financial difficulties, parents' relationship, family size, family major problems, maternal psychopathology, parents' substance abuse, crime records, partner support and social network. The FAI was assessed during pregnancy (long index), at 2 years (long index), and at 4 years (short index). The short index excludes social, practical, and financial support. Points were summed at each time point for a total FAI score across the 3 time points. Maternal socioeconomic status was measured using the Cambridge Social Interaction and Stratification Scale, which provides a total score. CI, confidence interval; Ethnicity, white vs non‐white; OR, Odd ratio.

**Figure 1:**
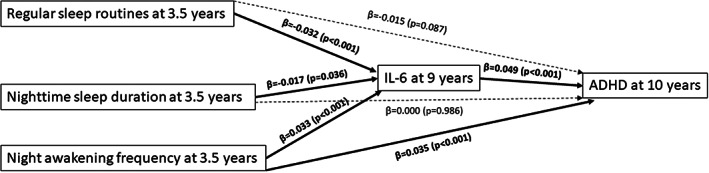
Path diagram showing the main direct associations, with IL‐6 at 9 years as mediating factor. This figure shows only the direct associations of the independent, mediating and dependent variable. Sleep variables (i.e., regular sleep routines, nighttime sleep duration and night awakening frequency) at 3.5 years represent the independent variables; IL‐6 at 9 years the mediating factor; and probable ADHD diagnosis at 10 years represents the outcome. The covariates also included in this path analyses were sex, family adversity and preterm delivery, due to the significant associations with the outcome found in the logistic regression model. Significant pathways are signified by solid arrows and nonsignificant pathways by dotted‐dashed lines

In relation to IL‐6 at 9 years as mediator of the association between early childhood sleep variables and probable ADHD diagnosis at 10 years, model fit indexes indicated excellent model fit (χ^2^ = 0.27, *p* = .60; RMSEA = 0; CIF = 1.00). Consistent with the adjusted logistic regression, higher night awakening frequency at 3.5 years was directly and significantly associated with probable ADHD diagnosis at 10 years (β = .035, *p* < .001). Direct associations are shown in Figure 1. Further, we observed an indirect effect of IL‐6 at 9 years in the association between regular sleep routines at 3.5 years and probable ADHD diagnosis at 10 years (β =−.002; 95% CI = −0.003 to −0.001, *p* = .005; 12.5% of the total effect was mediated by IL‐6 at 9 years); and between higher night awakening frequency at 3.5 years and probable ADHD diagnosis at 10 years (β = .002; 95% CI = 0.001 to 0.003, *p* = .003; 5.40% of the total effect was mediated by IL‐6 at 9 years) (see Table S4 for the indirect effects).

**Figure 2:**
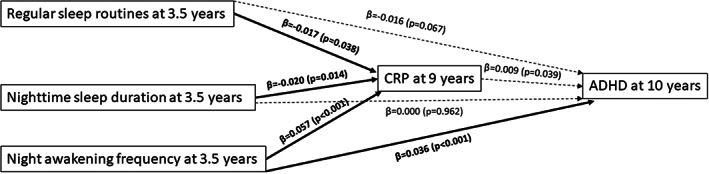
Path diagram showing the main direct associations, with CRP at 9 years as mediating factor. This figure shows only the direct associations of the independent, mediating and dependent variable. Sleep variables (i.e., regular sleep routines, nighttime sleep duration and night awakening frequency) at 3.5 years represent the independent variables; CRP at 9 years the mediating factor; and probable ADHD diagnosis at 10 years represents the outcome. The covariates also included in this path analyses were sex, family adversity and preterm delivery, due to the significant associations with the outcome found in the logistic regression model. Significant pathways are signified by solid arrows and nonsignificant pathways by dotted‐dashed lines

Concerning the mediating role of CRP at 9 years, we also demonstrated a good model fit (χ^2^ = 2.22, *p* = .14; RMSEA = 0.009; CIF = 1.00). Higher night awakening frequency at 3.5 years was the only early sleep problem which was directly and significantly associated with probable ADHD diagnosis at 10 years (β = 0.036, *p* < .001). Direct associations are shown in Figure 2. However, CRP at 9 years did not mediate any of the associations between early childhood sleep problems and probable ADHD diagnosis at 10 years (see Table S2 for the indirect effects).

We additionally controlled for sleep at 9 years (i.e., nighttime sleep duration) and probable ADHD diagnosis (measured with parent‐reported DAWBA) at 8 years, in the path analyses where IL‐6 at 9 years was included as mediator (i.e., the inflammatory marker with significant indirect effect). We controlled for these two additional variables to account for the potential impact of sleep at 3.5 years on sleep at 9 years, which subsequently could influence IL‐6 at 9 years, and to account for the potential impact of sleep at 3.5 years on ADHD at 8 years. We included these two variables (nighttime sleep duration at 9 years and probable ADHD diagnosis at 8 years) as covariates in the mediation analyses. Model fit indexes indicated excellent model fit (χ^2^ = 0.20, *p* = .66; RMSEA = 0; CIF = 1.00). Consistent with the results from the original path analyses with IL‐6 at 9 years as mediator, higher night awakening frequency at 3.5 years was directly and significantly associated with probable ADHD diagnosis at 10 years (β = .026, *p* < .001). Direct associations are shown in Figure S2. Further, we observed an indirect effect of IL‐6 at 9 years in the association between regular sleep routines at 3.5 years and probable ADHD diagnosis at 10 years (β = −.002; 95% CI = −0.003 to −0.001, *p* = .006, 16.7% of the total effect was mediated by IL‐6 at 9 years) and between higher night awakening frequency at 3.5 years and probable ADHD diagnosis at 10 years (β = .002; 95% CI = 0.001 to 0.003, *p* = .006; 3.7% of the total effect was mediated by IL‐6 at 9 years).

Finally, sensitivity analyses using PMS showed similar results for the logistic regression and the path analyses. Regarding logistic regression analyses, we observed that the same sleep variables at 3.5 years (i.e., regular sleep routines, nighttime sleep duration and night awakenings), which were significantly associated with probable ADHD diagnosis at 10 years in the adjusted model when no PMS was applied, still remained significant in the unadjusted model after applying PMS (see Table S3). Concerning path analyses, and after applying PMS, we still obtained an excellent model fit for IL‐6 at 9 years as a mediating factor (χ^2^ = 0.27, *p* = .60; RMSEA = 0; CIF = 1.00). Further, the same significant direct effects remained (see Figure S3), and we still obtained an indirect effect of IL‐6 at 9 years in the association between regular sleep routines at 3.5 years and probable ADHD diagnosis at 10 years (β = −.002; 95% CI = −0.003 to −0.001, *p* = .012); and between higher night awakening frequency at 3.5 years and probable ADHD diagnosis at 10 years (β = .002; 95% CI = 0.001 to 0.003, *p* = .012).

## Discussion

To our knowledge, this is the first longitudinal study to examine whether inflammation mediates the association between early childhood sleep problems and probable ADHD diagnosis at 10 years. First, we found that irregular sleep routines, shorter nighttime sleep duration and higher night awakening frequency at 3.5 years were all associated with probable ADHD diagnosis at 10 years. Second, we found that IL‐6 at 9 years mediated the associations of both irregular sleep routines and higher frequency of night awakening at 3.5 years with probable ADHD diagnosis at 10 years. In contrast, CRP at 9 years did not mediate any of the associations between early childhood sleep problems and probable ADHD diagnosis at 10 years.

We found that several parent‐reported sleep problems in early childhood were associated with probable ADHD diagnosis at 10 years. This is consistent with previous research supporting that sleep problems in early childhood constitutes an important risk factor for developing ADHD. For instance, parent‐reported short sleep duration at 5–6 years is prospectively associated with ADHD symptoms at 8–9 years (Tso et al., 2019). Furthermore, these prospective associations with short sleep duration have been also reported at earlier ages, with shorter sleep duration from 3 to 24 months being associated with inattention at 5 years (Huhdanpää et al., 2019). These prospective associations continue into adolescence, with recent evidence suggesting that several sleep problems in early childhood, including insomnia, restless leg syndrome and frequent snoring (Liu et al., 2020), as well as difficulty going to sleep, nightmares and restless sleep predict ADHD symptoms in adolescence (Carpena et al., 2020). Our findings contribute to the existing evidence by highlighting that short sleep length and sleep fragmentation in early childhood are also related to probable ADHD diagnosis at 10 years. Further, irregular sleep routines at 3.5 years were also related to probable ADHD diagnosis at 10 years, suggesting that a lack of routine is an important factor to consider with regard to ADHD, as has been previously reported (Weiss, Wasdell, Bomben, Rea, & Freeman, 2006).

Several underlying mechanisms have been suggested to account for the link between sleep and ADHD. For example, sleep deprivation can impact the prefrontal cortex (Owens et al., 2013) and weaker function and structure of prefrontal cortex circuits associate with ADHD (Arnsten, 2009). Further, dopaminergic activity is associated with ADHD symptomology (Solanto, 2002) and is also implicated in the regulation of sleep‐waking (Monti & Monti, 2007). Here, we focused on the two most commonly assayed inflammatory markers (i.e. IL‐6 and CRP) as potential mediators of the prospective associations between sleep and ADHD in childhood, due to a possible role of inflammation in ADHD pathogenesis (Anand, Colpo, Zeni, Zeni, & Teixeira, 2017).

Our initial hypothesis that both markers of inflammation would mediate this association was partially supported. We found that IL‐6, but not CRP, at 9 years, mediated the associations between irregular sleep routines and frequent night awakening at 3.5 years, with ADHD at 10 years. The associations of IL‐6 and CRP with subsequent outcomes, including mental health and brain development, may differ. For instance, in a longitudinal study, higher levels of IL‐6 in childhood, but not CRP, were associated with increased risk of depression and psychosis in young adulthood (Khandaker, Pearson, Zammit, Lewis, & Jones, 2014). Similarly, IL‐6 at 9 years, but not CRP, was prospectively associated with diurnal mood variation, concentration difficulties, fatigue and sleep disturbances at 18 years old (Chu et al., 2019) and with increased risk of hypomanic symptoms by age 22 (Hayes et al., 2017). A recent study also reported in a mendelian randomization analysis that genetic variants regulating levels and activity of IL‐6/IL6R, but not CRP, were associated with gray matter volume (especially in the middle temporal gyrus and fusiform gyrus), and with cortical thickness (mainly in the superior frontal gyrus) (Williams et al., 2022), which are areas implicated in ADHD.

So far, it is still unknown why IL‐6 but not CRP might play a greater role in the development of mental health problems. One potential explanation might be found in the different physiological actions associated with each of these inflammatory markers (Del Giudice & Gangestad, 2018). However, it is still unknown how this might lead to different adverse outcomes and under which circumstances. Therefore, further research to understand and compare the potential distinct role of IL‐6 and CRP in the development of mental disorders is needed.

This study has several strengths, including the large population‐based sample size, the longitudinal design and the inclusion of several sleep variables in early childhood. There are also some limitations. First, other potential contributing factors, such as depression, cognition, obstetric complications, viral infections, smoking or body mass index, which could have some effect on the results were left unexplored. For this study, we decided to control only for socio‐demographic factors and early life family adversities and not to over‐control for variables, consistent with our previous research using this cohort (Morales‐Muñoz et al., 2020; Morales‐Muñoz, Palmer, Marwaha, Mallikarjun, & Upthegrove, 2022); however, further relevant confounders should be also explored in future studies. Second, probable ADHD at 10 years was coded by collecting information from parents, and thus parental perceptions and/or assumptions could bias this assessment. Similarly, sleep was reported by the parents, and thus future studies should focus on self‐reported and objective sleep measures. Third, the time points included in this study were partially determined by the availability of the data collected in the ALSPAC, and thus other potentially relevant time points were left unexplored, which could have an impact on our results. Further, there was only one point for the markers' measure (e.g., one single measure of sleep at 3.5 years old), and this is a limitation as there are circadian variation of these markers' secretion. Fourth, as might be expected in a long‐term population cohort study, the attrition rate was significant, and this could also constitute a potential bias of our results. However, we used well established procedures to increase the representativeness in the form of inverse probability weighting method. This method assists in reducing bias in the estimates of the effect because it accounts (at least partially) for selection bias due to attrition. Finally, the prevalence of children with probable ADHD diagnosis using the top two levels of the DABWA‐bands as computer‐generated DAWBA diagnoses was considerably smaller (i.e., 1.6%) than the prevalence reported by previous studies (e.g., around 5%). These discrepancies might be related to the different methodology used to assess ADHD. However, the lower prevalence detected in our study supports the assumption that the DAWBA represents clinically relevant ADHD cases, and that is likely to only include those children with probable ADHD diagnosis. However, another potential explanation could be related to the characteristics of the DAWBA per se, as this has been noted to be a conservative measure, compared to other psychiatric interviews (Angold et al., 2012). Therefore, this should be taken into account when interpreting our results.

In summary, our findings showed that shorter nighttime sleep duration, higher night awakening and more irregular sleep routines at 3.5 years were associated with probable ADHD diagnosis at 10 years. Further, IL‐6 at 9 years, but not CRP mediated the associations between early sleep problems and probable ADHD diagnosis at 10 years. These findings support the relevant role of sleep problems in early childhood as a risk factor for ADHD. Further, a non‐resolving proinflammatory mechanism might be a contributory pathway explaining why sleep problems in early childhood are linked to subsequent ADHD, and thus provides further support to the role of inflammation in mechanistic pathways to ADHD. These results highlight the potential of future preventative interventions in ADHD, with the novel target of sleep and inflammation.

## Supporting information


**Appendix S1.** Further details of the ALSPAC cohort.
**Appendix S2.** Further details of the Development and Well‐Being Assessment (DAWBA).
**Table S1.** Differences in socio‐demographic variables between nonparticipating and participating subjects in the study at 10 years old.
**Table S2.** Pearson correlations between sleep variables at 3.5 years.
**Table S3.** Logistic regression analyses between sleep problems at 3.5 years and probable ADHD diagnosis at 10 years, after applying propensity matching score.
**Table S4.** Bootstrapped bias‐corrected confidence intervals and p values for the hypothesized indirect pathways to probable ADHD diagnosis at 10 years with IL6 at 9 years and CRP at 9 years as mediators.
