# Supplementary material for: The role of inflammation in the prospective associations between early childhood sleep problems and ADHD at 10 years: findings from a UK birth cohort study
Source: J Child Psychol Psychiatry. 2023 Jan 3;64(6):930–40. doi: 10.1111/jcpp.13755 (PMC10952536; doi:10.1111/jcpp.13755)

**Supporting Information**

**Appendix S1. Further details of the ALSPAC cohort:**

The initial number of pregnancies enrolled was 14,541 (for these at least one questionnaire was returned, or a “Children in Focus” clinic had been attended by 19/07/99). Of these initial pregnancies, there was a total of 14676 foetuses, resulting in 14062 live births and 13988 children who were alive at 1 year of age. When the oldest children were approximately 7 years of age, an attempt was made to bolster the initial sample with eligible cases who had failed to join the study originally. As a result, in our study, as some variables were collected from the age of seven onwards there were data available for more than the 14541 pregnancies mentioned above. Informed consent for the use of data collected via questionnaires and clinics was obtained from participants following the recommendations of the ALSPAC Ethics and Law Committee at the time. Ethical approval was obtained from the ALSPAC Law and Ethics committee and the local research ethics committees.

**Appendix S2. Further details of the Development and Well-Being Assessment (DAWBA):**

The DAWBA is a package of interviews, questionnaires and rating techniques designed to generate ICD-10 and DSM-IV or DSM-5 psychiatric diagnoses about 2-17 years old. The DAWBA includes a mix of ‘closed’/structured questions and open-ended questions, where respondents describe their difficulties in their own words. The full DAWBA package covers the following diagnoses: Separation anxiety, Specific phobia, Social phobia, Panic disorder/agoraphobia, Post-traumatic stress disorder, Obsessive compulsive disorder, Generalized anxiety disorder, Body dysmorphic disorder, Disruptive mood dysregulation disorder, Major depression, ADHD/hyperkinesis, Oppositional defiant disorder, Conduct disorder, Eating disorders, including anorexia, bulimia and binge eating, Autism spectrum disorders, Tic disorders, including Tourette syndrome, and Bipolar disorders. For each of these disorders, the interview asks about all the symptoms, and other criteria needed for an operationalized diagnosis according to both DSM-IV (American Psychiatric Association, 1994) and the research diagnostic version of ICD-10 (World Health Organisation, 1994). Panic disorder, agoraphobia, autistic disorders, eating disorders, tic disorders, and any other concerns are covered more briefly, with clinical diagnoses of these disorders being correspondingly more dependent on rating the open-ended transcript. The time frame of the interview is the present and the recent past. For many disorders, the ICD-10 and DSM-IV diagnostic criteria stipulate that the symptoms need to have persisted for a specified number of months, e.g., a minimum of 6 months for hyperactivity, oppositional-defiant disorder, and generalized anxiety disorders. In these instances, the relevant section of the DAWBA interview focuses on the child’s symptoms over this

stipulated period. The time frame is longest for conduct disorder (since DSM-IV criteria include the number of relevant behaviours displayed over the previous 12 months), and shortest for most of the emotional disorders, where the focus is on the last month, in line with previous recommendations (Shaffer et al., 1996).

**Table S1.** Differences in socio-demographic variables between non-participating and participating subjects in the study at 10 years old

|  | **Non-participating group in the study** | | **Participating group in the study** | | | **Non-participating versus participating** | |
| --- | --- | --- | --- | --- | --- | --- | --- |
|  | *Mean* | *SD* | *Mean* | *SD* | | *OR (95% CI)* | *p* |
| Maternal age when born | 26.81 | 5.11 | 29.05 | 4.58 | | 1.10 (1.09 to 1.11) | <0.001 |
| Gestational age | 37.24 | 7.46 | 39.45 | 1.85 | | 1.14 (1.10 to 1.13) | <0.001 |
| Birth weight, kg | 3.34 | 0.62 | 3.42 | 0.54 | | 1.28 (1.21 to 1.35) | <0.001 |
| Family Adversity score | 5.34 | 4.75 | 3.93 | 4.01 | | 0.93 (0.92 to 0.94) | <0.001 |
|  | **Non-participating group in the study** | | **Participating group in the study** | | |  |  |
|  | *N* | *%* | *N* | | *%* |  |  |
| Sex  Male / Female | 3772 / 3498 | 51.9 / 48.1 | 3919 / 3850 | | 50.4 / 49.6 | 0.94 (0.88 to 1.01) | 0.077 |
| Ethnicity |  |  |  | |  | 0.42 (0.33 to 0.52) | <0.001 |
| White / Other | 4985 / 205 | 96.1 / 3.9 | 7077 / 1.7 | | 98.3 / 1.7 |  |  |
| Preterm delivery |  |  |  | |  | 0.56 (0.49 to 0.65) | <0.001 |
| Yes / No | 509 / 3443 | 12.9 / 87.1 | 347 / 4169 | | 7.7 / 92.3 |  |  |

The individuals associated with attrition at 10 years were more frequently born preterm, had lower weight at birth, had shorter gestational age, had younger mothers at birth, had higher scores in family adversity, and were more often of non-white ethnicity.

**Table S2.** Pearson correlations between sleep variables at 3.5 years

|  | Regular sleep routines | Daytime sleep duration | Nighttime sleep duration | Number of night awakening |
| --- | --- | --- | --- | --- |
| Regular sleep routines | ------------- | r=-0.131, p<0.001 | r=-0.273, p<0.001 | r=-0.244, p<0.001 |
| Daytime sleep duration | r=-0.131, p<0.001 | ------------- | r=0.222, p<0.001 | r=0.011, p=0.356 |
| Nighttime sleep duration | r=-0.273, p<0.001 | r=0.222, p<0.001 | ------------- | r=-0.157, p<0.001 |
| Number of night awakening | r=-0.244, p<0.001 |  | r=-0.157, p<0.001 | ------------- |

**Table S3.** Logistic regression analyses between sleep problems at 3.5 years and probable ADHD diagnosis at 10 years, after applying propensity matching score.

| **Probable ADHD diagnosis at 10 years** | | | |
| --- | --- | --- | --- |
|  | **Unadjusted model** | | |
|  | OR | 95% CI | p value |
| Regular sleep routines, 3.5 years | **0.52** | **0.27 to 0.99** | **0.050** |
| Daytime sleep duration, hours, 3.5 years | 1.22 | 0.94 to 1.57 | 0.129 |
| Nighttime sleep duration, hours, 3.5 years | **0.73** | **0.58 to 0.92** | **0.007** |
| Night awakenings per night, 3.5 years | **1.24** | **1.02 to 1.51** | **0.029** |

*Individual propensity scores were calculated through logistic regression modelling based on the following 8 covariates: sex, FAI, preterm delivery, ethnicity, maternal age at child’s birth, maternal socio-economic status, maternal tobacco consumption during pregnancy and child’s intelligence quotient. The “unexposed” and “exposed” cases were then paired 5:1 nearest neighbour matching.

**Table S4.** Bootstrapped bias-corrected confidence intervals and p values for the hypothesized indirect pathways to probable ADHD diagnosis at 10 years with IL6 at 9 years, and CRP at 9 years as mediators

|  | Probable ADHD diagnosis at 10 years | | | | Probable ADHD diagnosis at 10 years | | | |
| --- | --- | --- | --- | --- | --- | --- | --- | --- |
|  | Via IL-6 at 9 years | | | | Via CRP at 9 years | | | |
|  | β | Lower Bound | Upper Bound | p | β | Lower Bound | Upper Bound | p |
| Nigh awakening frequency 3.5 years | **0.002** | **0.001** | **0.003** | **0.003** | 0.000 | -0.001 | 0.000 | 0.329 |
| Regular sleep routines 3.5 years | **-0.002** | **-0.003** | **-0.001** | **0.005** | 0.000 | -0.001 | 0.000 | 0.268 |
| Night sleep duration 3.5 years | -0.001 | -0.002 | 0.000 | 0.093 | 0.000 | 0.000 | 0.001 | 0.365 |

IL-6=Interleukin 6; CRP=C-reactive protein


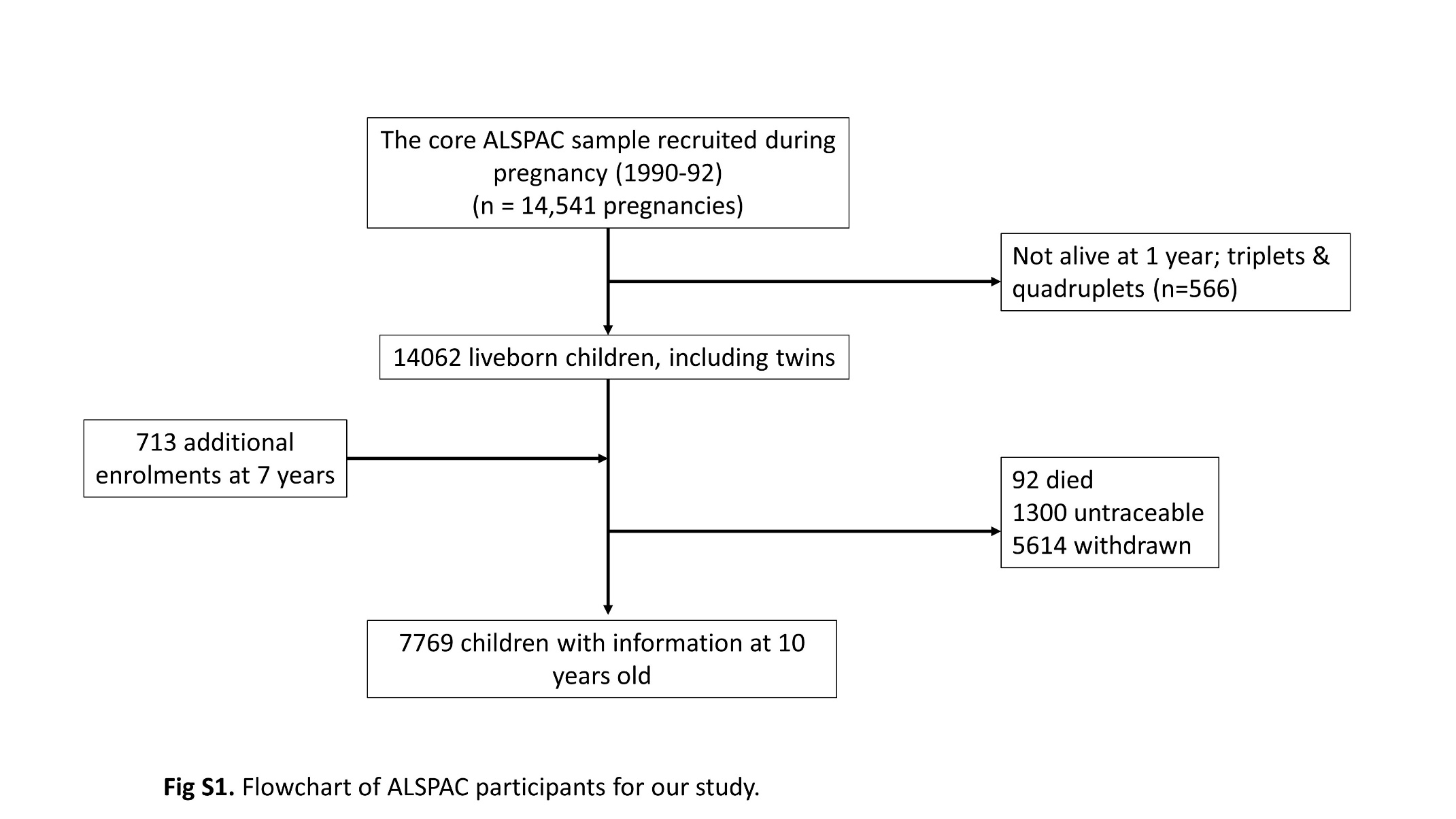


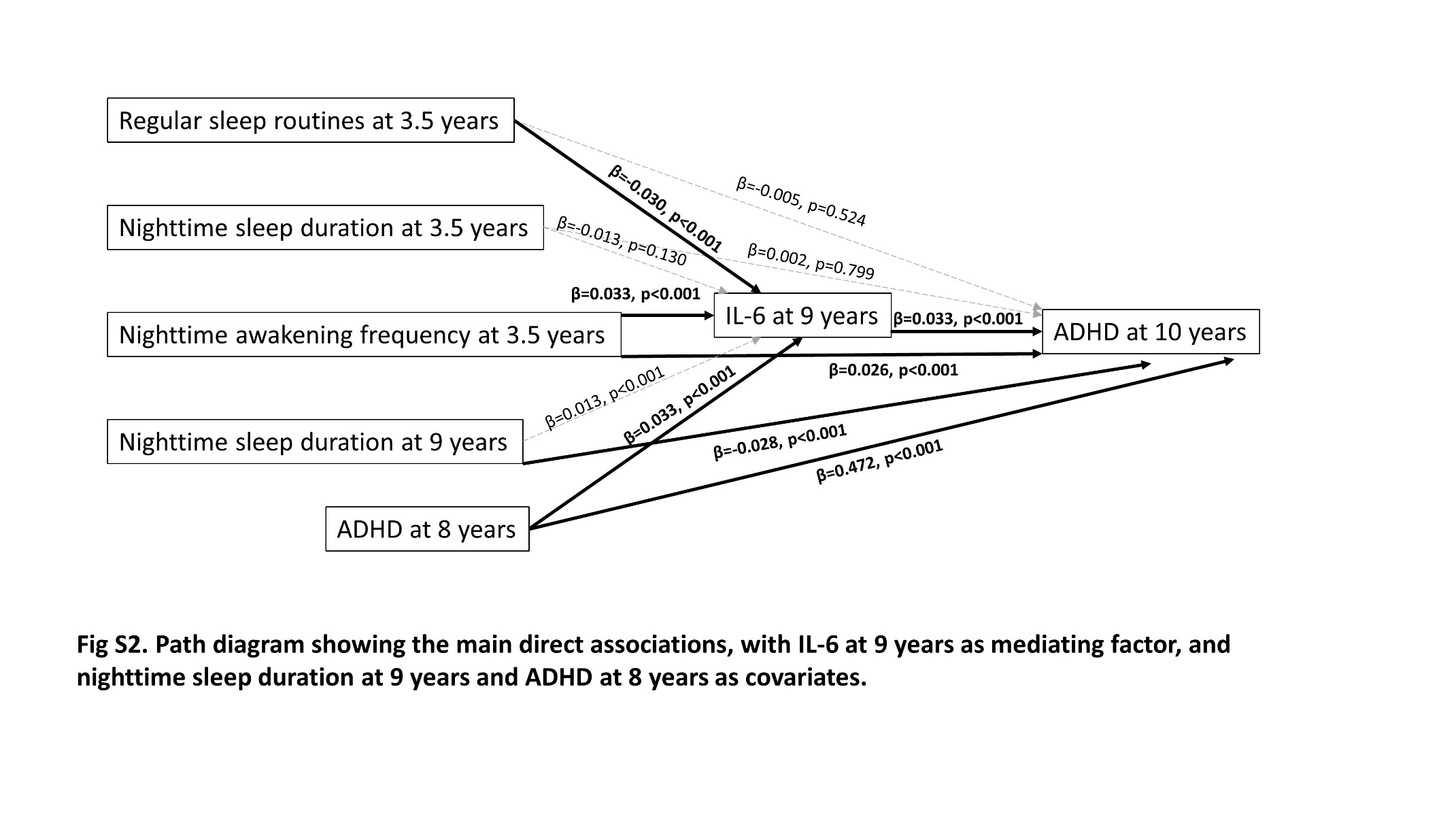


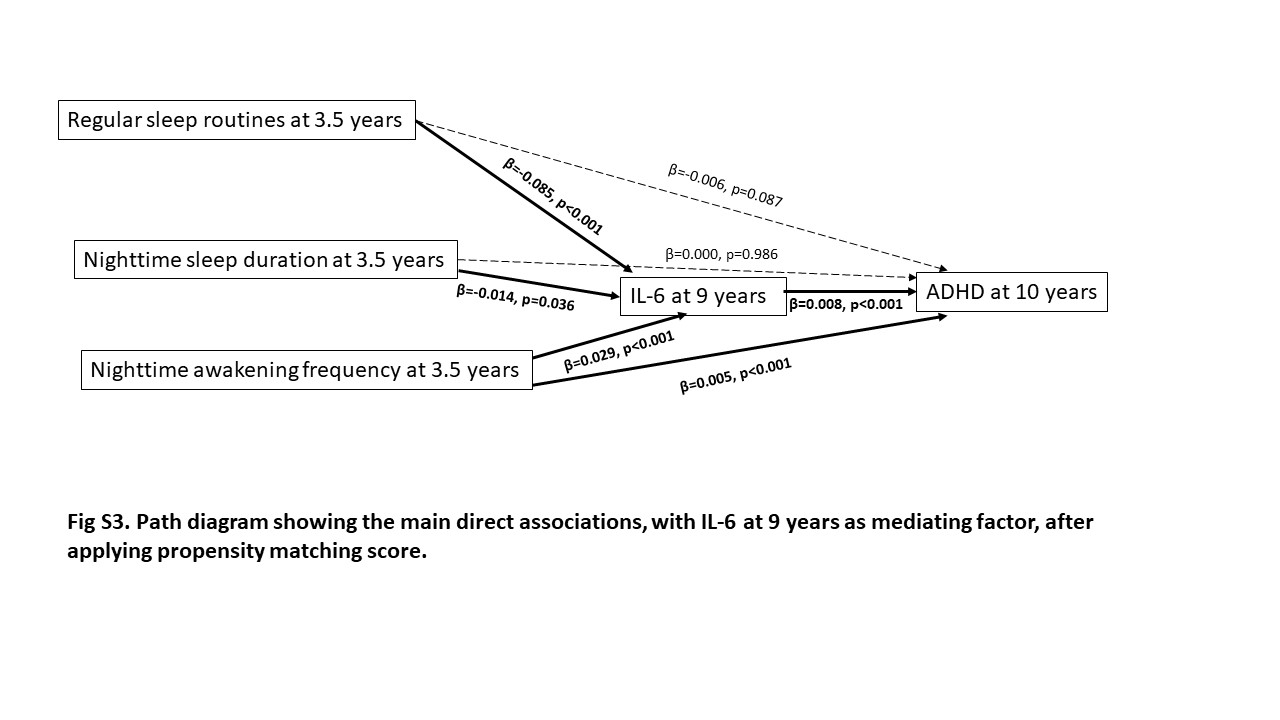

Supplement: Supplementary file 1 — Appendix S1. Further details of the ALSPAC cohort. Appendix S2. Further details of the Development and Well‐Being Assessment (DAWBA). Table S1. Differences in socio‐demographic variables between nonparticipating and participating subjects in the study at 10 years old. Table S2. Pearson correlations between sleep variables at 3.5 years. Table S3. Logistic regression analyses between sleep problems at 3.5 years and probable ADHD diagnosis at 10 years, after applying propensity matching score. Table S4. Bootstrapped bias‐corrected confidence intervals and p values for the hypothesized indirect pathways to probable ADHD diagnosis at 10 years with IL6 at 9 years and CRP at 9 years as mediators. [file JCPP-64-930-s001.docx]
